# Selective Supercritical CO_2_ Extraction and Biocatalytic Valorization of *Cucurbita pepo* L. Industrial Residuals

**DOI:** 10.3390/molecules27154783

**Published:** 2022-07-26

**Authors:** Alessio Massironi, Alessandro Di Fonzo, Ivan Bassanini, Erica Elisa Ferrandi, Stefania Marzorati, Daniela Monti, Luisella Verotta

**Affiliations:** 1 Department of Environmental Science and Policy, Università degli Studi di Milano, Via Celoria 2, 20133 Milano, Italy; stefania.marzorati@unimi.it (S.M.); luisella.verotta@unimi.it (L.V.); 2 Istituto di Scienze e Tecnologie Chimiche “Giulio Natta”, Consiglio Nazionale delle Ricerche, Via Mario Bianco 9, 20131 Milano, Italy; alessandro.difonzo@scitec.cnr.it (A.D.F.); ivan.bassanini@cnr.it (I.B.); erica.ferrandi@scitec.cnr.it (E.E.F.)

**Keywords:** supercritical CO_2_, biomass valorization, biocatalysis, fatty acids, *Cucurbita pepo* L.

## Abstract

The valorization of biomass residuals constitutes a key aspect of circular economy and thus a major challenge for the scientific community. Among industrial wastes, plant residuals could represent an attractive source of bioactive compounds. In this context, a residue from the industrial extraction of *Cucurbita pepo* L. seeds, whose oil is commercialized for the treatment of genito-urinary tract pathologies, has been selected. Supercritical CO_2_ technology has been employed as a highly selective “green” methodology allowing the recovery of compounds without chemical degradation and limited operational costs. Free fatty acids have been collected in mild conditions while an enrichment in sterols has been selectively obtained from sc-CO_2_ extracts by appropriate modulation of process parameters (supercritical fluid pressure and temperature), hence demonstrating the feasibility of the technique to target added-value compounds in a selective way. Obtained fatty acids were thus converted into the corresponding ethanol carboxamide derivatives by lipase-mediated biocatalyzed reactions, while the hydroxylated derivatives of unsaturated fatty acids were obtained by stereoselective hydration reaction under reductive conditions in the presence of a selected FADH_2_-dependent oleate hydratase.

## 1. Introduction

Plants represent an inestimable source of bioactive compounds for humans. From ancient times, humankind has always exploited the vegetal variability for a widespread number of applications spanning from their use as food, textile manufacture, and for the cure of several diseases, posing the basis of modern medicine [1,2]. Starting from the simple ingestion or topical applications of plant parts, the scientific progression allowed understanding of the bioactive agents involved in the disease treatments, finally becoming an inspiration source for the synthesis of new drugs [3,4]. Most of their unique properties are ascribable to plant bioactives, which are commonly produced by the secondary metabolism, displaying a pharmacological or, depending on their application, toxicological response in humans [5,6]. As a consequence, there is a growing interest in the nutraceutical industry, as well as the research of new bioactive molecules and techniques to easily extract and isolate them [7,8,9,10]. Such increments in plant research have in parallel led to a rapid accumulation of biomass residues, requiring the development of sustainable procedures for their valorization [11]. Indeed, millions of tons of biomass are annually generated from agriculture and health food industries worldwide, causing several waste governance and disposal issues [12]. In this context, their possible recirculation as a further source of molecules with biological and industrial interests represents a major challenge for the scientific community [13].

In the present research, a residue from a selective industrial supercritical CO_2_ extraction of *Cucurbita pepo* L. (Cp) seeds, whose oil is commercialized for the treatment of genito-urinary tract pathologies, has been selected for its possible exploitation as a novel low-cost source of bioactive agents. Cp residues have been provided by Indena S.p.A. (https://www.indena.com, accessed on 28 June 2022), a leading company in the identification, development, and production of plant-derived active principles for the pharmaceutical and health food industries.

Pumpkin (Cp) seed lipophilic extracts are rich sources of several bioactive compounds and are commonly used as edible oil or as potential nutraceutical ingredients [14]. The literature reports 50 to 80% oil content in pumpkin seeds, composed of fatty acids in a percentage from 70 to 80.5% with a predominant presence of palmitic (C16), stearic (C18), oleic (C18:1), and linoleic acids (C18:2) [15]. To date, different studies have confirmed the medical properties of pumpkin seed oil. Its unique bioactivity seems related to ∆7 sterols, providing protection against hypertension and carcinogenic diseases [14]. Moreover, oils from Cp seeds are used in combination with *Serenoa repens* fruit extracts for the treatment of the same pathologies and to prevent prostatic cancer [16,17].

The investigated Cp seeds were provided after an industrial supercritical CO_2_ (sc-CO_2_) extraction process by the company. Given the selectivity of the technology, it was considered likely that some unextracted compounds were still present in the residues to be further processed. With this aim, the presence of residual compounds in Cp residue of potential biological and industrial interest was investigated. Focused attention has been devoted to two classes of compounds, namely, ∆7-sterols and fatty acids [15]. In fact, in contrast to the other vegetable oils with Δ5-sterols as the major components, different studies demonstrated that pumpkin seed oil contains specific Δ7-phytosterols, typical of only few plant families (e.g., Cucurbitaceae). Sterols commonly composed up to 70% of the total unsaponifiable matter of Cp seed oil [14,18]. Δ7-sterols represent important high-value compounds since are one of the key active components in the treatment of benign prostatic hyperplasia [19]. The chemical nature of selected compounds suggested their extractability in supercritical CO_2_, without the need for a co-solvent as a polarity modifier. Supercritical fluid technology represents a promising green alternative to conventional solvents for the extraction of non-polar molecules. Moreover, it is known that different CO_2_ densities allow a selective extraction of targeted species during the extraction through a simple tuning of temperature and pressure conditions [20,21,22,23]. Indeed, the correct tuning and optimization of these parameters, during the extraction process, represents a fundamental aspect for the development of the methodology that allows selective enrichment, depending on the target compounds [7].

Aiming to define the presence of compounds of interest still available in the residues, in this work sequential selective extractions, at different and increasing CO_2_ densities, have been carried out in order to screen the potential windows of selectively extractable molecules. Obtained fractions were characterized initially through a main composition analysis where the amount of free fatty acids with respect to neutral components was evaluated. Second, the unsaponifiable matter of obtained fractions has been quantified and then characterized through gas chromatography–mass spectrometry (GC-MS).

The sc-CO_2_ fractions obtained through sequential extraction composition have been evaluated and compared to: (i) a single-step sc-CO_2_ extraction, carried out by applying the strongest conditions (in terms of pressure and temperature, and hence CO_2_ density), and (ii) a conventional *n*-hexane extract.

Fractions enriched in fatty acids were then subjected to biocatalyzed manipulations leading to the preparation of selected ethanolamide and hydrated derivatives, both products finding a wide range of applications in different fields. Specifically, members of the fatty acid ethanolamide family, such as arachidonylethanolamide (AEA, also known as anandamide), oleoylethanolamide (OEA), and palmitoylethanolamide (PEA), show a variety of biological activities, e.g., anti-inflammatory, anorexic effects—useful for appetite control in people with obesity—and analgesic effects [24,25]. On the other side, hydroxy fatty acids (HFAs), obtained by hydration of unsaturated fatty acids (UFAs), are important precursors of flavors, biodegradable polymers, lubricants, cosmetic ingredients, and emulsifiers [26,27,28,29]. Both enzymatic reactions were first optimized by selection of the respective biocatalyst and biotransformation conditions using standard fatty acids as substrates, then applied to the fatty acid-enriched Cp extracts.

## 2. Results and Discussion

### 2.1. sc-CO_2_ Extraction Rationale

Aiming to compare the successive sc-CO_2_ Cp extracts’ main composition and extraction yields, a solvent-based *n*-hexane extraction was first carried out on Cp biomass residuals to be employed as a reference.

Then, in order to identify the structural richness available in the residues and extractable by means of supercritical CO_2_, a single-step extraction was performed to achieve the complete biomass exhaustion by applying strong extraction conditions in terms of temperature (50 °C) and pressure (380 bar). Higher temperatures were not considered to preserve the chemical stability of all species contained in the residual plants and to avoid any kind of thermal degradation of compounds [30].

Finally, during the selective extractions, different sets of pressures and temperatures were chosen in accordance with the results obtained in the single-step sc-CO_2_ process. Three different CO_2_ densities, corresponding to mild (35 °C; 100 bar; 700 kg/m^3^), medium (45 °C; 220 bar; 830 kg/m^3^), and strong (equal to the single step: 50 °C; 380 bar; 913 kg/m^3^) conditions were identified as promising to study a potential achievable selectivity.

#### *Cucurbita pepo* L. Seed Waste Extraction

**Figure 1:**
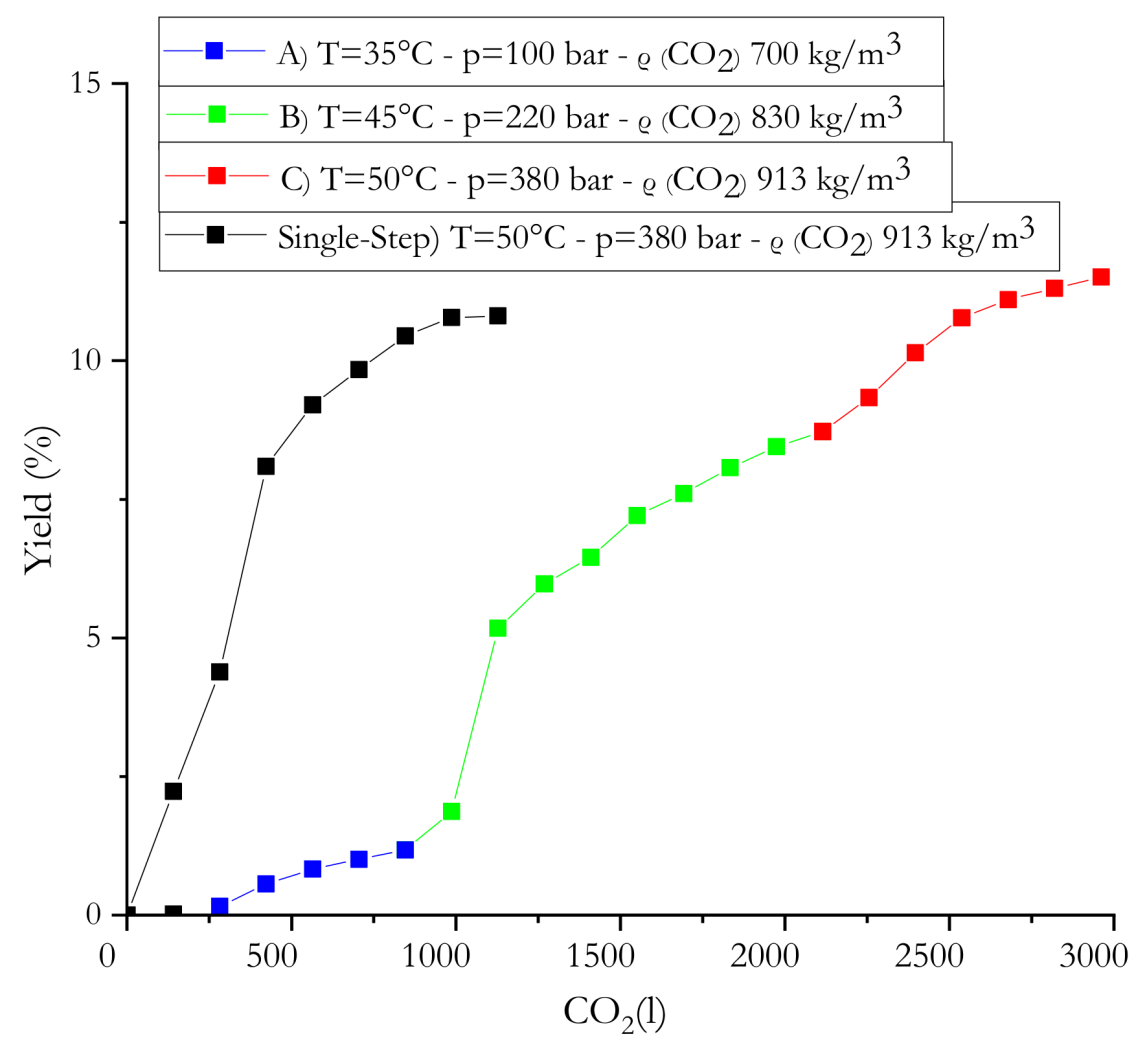
Extraction kinetics in single-step and sequential sc-CO_2_ extractions from Cp biomass.

Cp residuals, extracted through the mild conditions, gave a cumulative yield of about 1.2% (Cp_sc-CO_2_A, blue line in Figure 1), the second (medium) extraction increased the cumulative extraction yield up to 8.7% (Cp_sc-CO_2_B, green line in Figure 1), and, finally, in the strongest extraction conditions, an 11.5% yield was achieved (Cp_sc-CO_2_C, red line in Figure 1). The overall extraction curves are reported in Figure 1 in comparison to extraction kinetics achieved by means of single-step extraction (black line in Figure 1). The second set of parameters, carried out at 45 °C and 220 bar, were the most effective in terms of extraction yield gain (green line in Figure 1).

All the extraction kinetics (single-step and sequential extractions) follow the model explained by Patel et al., where an initial period of extraction (the steepest) represents the depletion of extractables found on the particle surface, while the final period represents the extraction of compounds from inside the porosity, where the mass transfer is more hindered and the extraction rate slows down until it zeroes [31,32].

Ion exchange chromatography was performed on each extract (solvent-based, single-step, and each of the sequential CO_2_ extracts) to separate free fatty acids from neutral compounds. Afterwards, neutral fractions were subjected to saponification to isolate the unsaponifiable matter. The main composition of extracts from Cp residuals is reported in Table 1.

As expected, single-step and the overall three-step extraction yields were similar since both methods lead to a complete exhaustion of the extractables contained in the processed biomass. However, by comparing the compositions, in terms of free fatty acids and neutral components as displayed in Table 1, the critical role of CO_2_ density, affecting the solutes solubility, was observed. In fact, changes in temperature and pressure parameters led to completely different extractable enrichments. Analyzing results from sequential sc-CO_2_ extraction, the mild extraction condition (entry 2 in Table 1) allowed to obtain from Cp residuals a fraction enriched in free fatty acids (71%). As a consequence of their exhaustion, the percentage of fatty acids in the extract obtained in medium conditions fell to 24%, with a parallel increase in the unsaponifiable fraction from less than 0.5% (entry 2) to 4.1% (entry 3); finally, running the extraction in the strongest conditions, a fraction enriched in neutral components (96%, mostly composed of triglycerides) was obtained (entry 4).

Another reference is provided by the results of the composition of the *n*-hexane extract (entry 5). It displayed a similar extraction yield in comparison to single-step and sequential sc-CO_2_ extractions, but the percentage of neutral compounds was comparable with the single-step extract (entry 1). This result is reasonable since both solvent-based extraction and single-step extractions are meant to exhaust the extractable content without any selectivity.

### 2.2. Extract Characterization

To identify the compounds extracted from the residual biomass, the acidic fraction retained on Amberlite IRA-400 resin (composed of free fatty acids), and the unsaponifiable matter of collected extracts were characterized through GC-MS. Extracts obtained from sc-CO_2_ technology were compared to conventional solvent-based extraction and to literature data.

#### 2.2.1. *Cucurbita pepo* L. Acid Fraction Compositions

The relative abundances of isolated free fatty acids, separated through ion exchange resin, of single-step sc-CO_2_, Cp_sc-CO_2_A (A), Cp_sc-CO_2_B (B) and Cp_sc-CO_2_C (C) were similar and are reported in Table 2 with the standard deviation of triplicate analyses. These values are also comparable to fatty acid composition of *n*-hexane extract.

Finally, the percentages of monounsaturated fatty acids (MUFAs), polyunsaturated fatty acids (PUFAs), and saturated fatty acids (SFAs) are reported in Table 2; again, no differences have been observed between *n*-hexane and sc-CO_2_ extracts. This result is probably due to the low polarity differences among extractable fatty acids, which did not allow their selective accumulation by applying different CO_2_ densities.

Among obtained extracts, Cp_sc-CO_2_A presented the highest content of free fatty acids (equal to 71%, entry 2, Table 1), making it a sustainable candidate source of free fatty acids from residual biomass. It is worth highlighting that the adopted temperature and pressure for Cp_sc-CO_2_A were significantly mild, in comparison to common CO_2_ extractions adopted for Cp commercial oils, where stronger extraction conditions may ensure higher yields, but at the expense of low extraction selectivity in terms of free fatty acids [33,34]. Free fatty acids represent in fact a class of compounds with widespread fields of application such as the pharmaceutical, cosmetic, and food industries [35]. Their possible extraction and selective accumulation from biomass residues represent a promising and sustainable alternative.

#### 2.2.2. *Cucurbita pepo* L. Unsaponifiable Fraction Compositions

**Figure 2:**
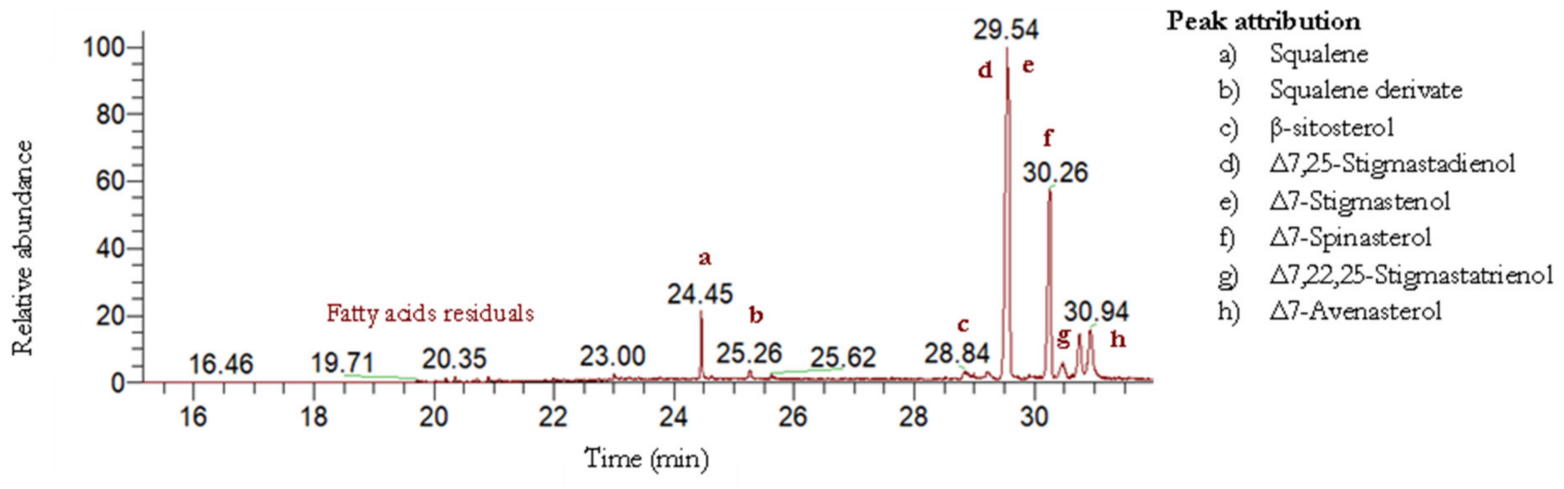
Total ion current and peak attributions of unsaponifiable fraction of Cp_sc-CO_2_.

Neutral Cp extract components were subjected to saponification reaction in methanolic KOH. The unsaponifiable matter was extracted in diethyl ether, while fatty acids were analyzed through GC-MS as methyl esters, presenting almost identical compositions to free fatty acids previously analyzed in the acidic fraction retained on the IRA 400 resin. Analysis of the unsaponifiable matter isolated from Cp residuals was performed through GC-MS, and the chromatogram and peak attributions are reported in Figure 2. Prior to the analysis, trimethylsilyl (TMS) ethers were obtained by derivatization through N,O-bis[trimethylsilyl]trifluoroacetamide (BSTFA). The trimethylsilyl ethers of the sterols were identified by comparison with the NIST mass spectra library via detection of the parent molecular ions [M]^+^ and fragmentation patterns of corresponding TMS derivatives; their fragmentations are listed in Table 3. β-Sitosterol and Δ7-stigmastenol were discriminated by injection of corresponding standards.

The results of GC-MS analysis confirmed the presence of Δ7-sterols in the extract, where only the β-sitosterol was observed for the Δ5 class, in particular; Δ7-spinasterol, Δ7-stigmastenol and Δ7,25-stigmastadienol composed almost 90% of the total sterol content.

Spectral data of Δ7-spinasterol, Δ7,25-stigmastadienol, Δ7-avenasterol having the same molecular masses (*m*/*z* = 484) were discerned by their main fragment ions: Δ7-spinasterol TMS ether was confirmed via its fragmentation ions derived from the loss of ethyl (*m*/*z* = 455) and *iso*-propyl (*m*/*z* = 441) groups, Δ7,25-stigmastadienol TMS ether was identified by the loss of the sole ethyl group (*m*/*z* = 455). Δ7-avenasterol TMS ether was finally identified by the absence of both fragmentation ions and confirmed with a certainty of 98% according to the NIST library. Other main fragment ions that allowed us to identify the single sterol were [M−90]^+^ and [M−105]^+^, which correspond to the loss of trimethylsilanol and the methyl group plus trimethylsilanol, respectively. The relative abundances of revealed species are reported in Table 3 and were calculated as relative area % detected under each peak.

In our results, the obtained unsaponifiable fraction was almost exclusively composed of sterols with traces of tocopherols, squalene derivatives, and carotenoids such as lutein. Traces of minor compounds such as γ-tocopherol and squalene were observed. No differences in terms of sterol composition were observed in the extracts from sc-CO_2_ sequential and single-step and *n*-hexane extracts [14,19].

### 2.3. Enzymatic Manipulation of Cucurbita pepo L. Extracts

The biocatalytic preparation of fatty acid ethanol amides exploiting lipase catalysis is a well-documented green synthetic entry to fine chemicals [45,46,47]. Different combinations of lipases, fatty acids, and reaction media (solvents, biocatalysts’ formulations, etc.) have been applied in biocatalytic amidations or two-step aminolysis [48,49,50,51,52], working on purified fatty acids of biological interest [53] or, in rare cases, directly exploiting simple plant oil extracts [54].

**Scheme 1:**
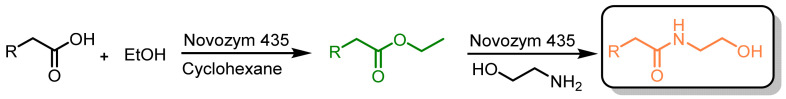
Two-step lipase-catalyzed synthesis of fatty acid ethanol amides.

As a proof of concept, after some preliminary screening of reaction conditions with commercially available fatty acids as substrates (data not shown), the Cp_sc-CO_2_A extract showing the highest content of free fatty acids was subjected to a two-step Novozym^®^ 435-catalyzed amidation protocol in which the fatty acid components are at first activated as ethyl esters and then converted into the desired ethanol amides by aminolysis (Scheme 1).

**Figure 3:**
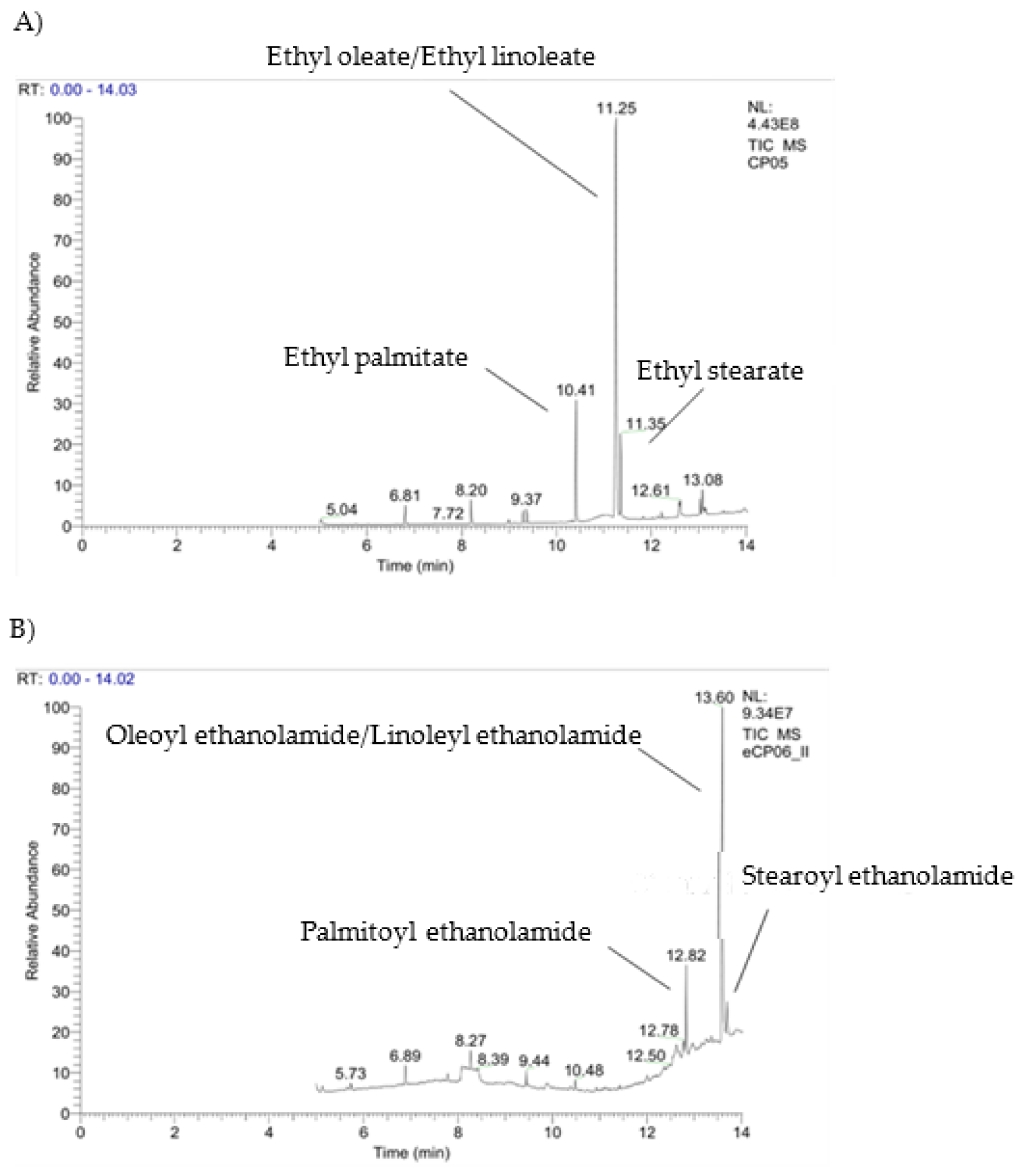
GC-MS chromatogram of the Novozym^®^ 435-catalyzed preparation of (**A**) ethyl esters and (**B**) ethanol amides of oleoyl, linoleoyl, stearyl, and palmitoyl acids in Cp extracts.

Briefly, the esterification step was conducted using a 10% (*v*/*v*) solution of ethanol in cyclohexane in the presence of 2 g L*^−^*^1^ of enzyme at 55 °C for 1 h. After recovering the mixture of fatty acid ethyl esters by filtrating the resin-supported biocatalyst and concentrating in vacuo, the desired ethanol amides were obtained in quantitative yields and conversion by reacting the recovered materials (45 g L*^−^*^1^) with ethanolamine, used as solvent, again in the presence of 2 g L*^−^*^1^ Novozym^®^ 435. GC-MS analysis confirmed the complete conversion of the starting fatty acid mixture into the corresponding ethanol amides within 72 h (Figure 3). For completeness of information, in the SI (Supplementary Material) are reported the GC-MS analyses of the biocatalytic esterification and amidation of palmitic acid (used for optimization) and of the ethanol amides of oleic and linoleic acids prepared as standards.

**Figure 4:**
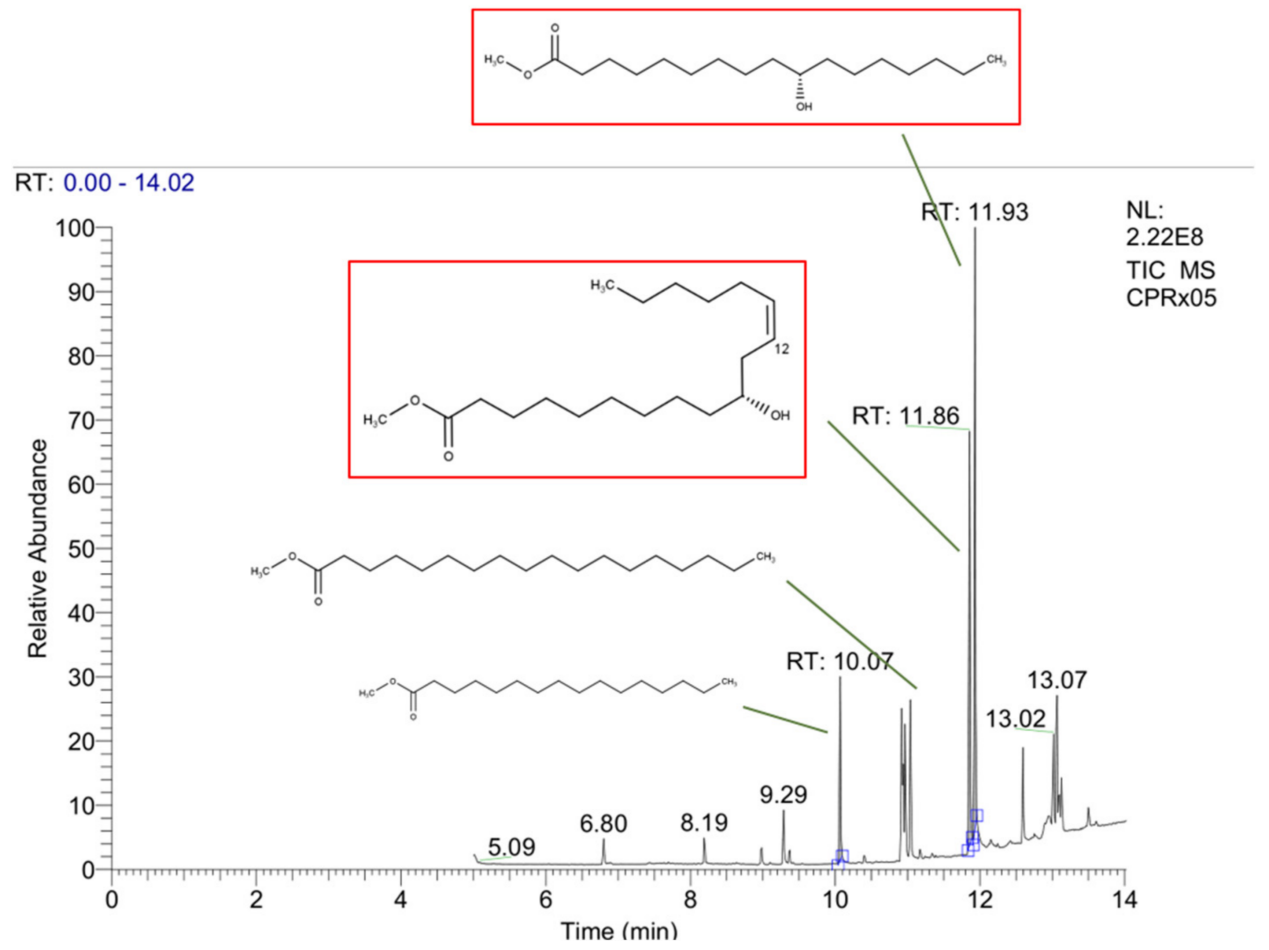
Biocatalyzed hydration of the unsaturated fatty acids oleic acid and linoleic acid in Cp extracts catalyzed by the oleate hydratase OhyA2.

In contrast to long-chain aliphatic acids, unsaturated fatty acids (UFAs) possess the potential to be converted into interesting derivatives in virtue of their reactive C-C double bond(s). UFAs can in fact be used to produce short-chain carboxyl acids of industrial or pharmaceutical interest [55,56,57] and, for this reason, the exploitation of green and sustainable chemo-enzymatic routes for their regio- and stereoselective hydration is highly cherished. In this context, the Cp_sc-CO_2_A fraction was also subjected to a biocatalyzed hydration exploiting the activity of the recombinant oleate hydratase from *Stenotrophomonas maltophilia* (OhyA2), an enzyme showing high activity as well as a broad substrate scope [58]. Similarly to other oleate hydratases [28,59,60], OhyA2 showed excellent performances when used in a reducing environment. Accordingly, Cp oil (20 g L*^−^*^1^) was incubated with OhyA2 (0.1 g L*^−^*^1^) in the presence of Ti^III^-citrate as reducing agent for 24 h at 35 °C, thus promoting the complete conversion of the two most abundant UFAs, i.e., oleic acid and linoleic acid (Table 2), into the corresponding 10-hydroxy derivatives (Figure 4).

## 3. Materials and Methods

### 3.1. Materials

After selective extraction of commercial oils by means of supercritical fluids, *Cucurbita pepo* L. (Cp) exhausted seeds were generously donated by Indena S.p.A. Carbon dioxide (CO_2_) was purchased from Sapio s.r.l (Monza, Italy) with a purity of 99.999%. BSTFA-TMCS (99:1) was from MACHEREY-NAGEL. HPLC grade and analytical grade acetonitrile, water, formic acids, Amberlite IRA-400, and beta-sitosterol standard were purchased from Sigma-Aldrich Chemicals (Milan, Italy). *n*-Hexane was purchased from Carlo Erba.

### 3.2. Cucurbita pepo L. Residual Extractions

#### 3.2.1. *n*-Hexane Residual Extraction

Twenty grams of Cp biomass residual were added to 80 mL of *n*-hexane in a 250 mL round flask and extracted under mechanical stirring at room temperature. After 6 h, the hexane phase was removed and an equal volume of fresh solvent was added to the biomass to continue the extraction procedure under the same conditions. In order to remove mucilage and coarse materials, the suspension was centrifuged at room temperature by using a rotational speed of 6000 rpm for 10 min. The extract was concentrated by a rotary evaporator (final extract weight of 2.0 g).

##### 3.2.2. Supercritical CO_2_: Single-Step Extraction

Supercritical fluid extractions were performed using a pilot unit SFT110XW System supplied by Supercritical Fluid Technologies, Inc. (Newark, DE, USA). It consisted of a 100 cm^3^ stainless steel extractor inserted in an oven, a constant pressure piston pump (SFT-Nex10 SCF Pump) with a Peltier cooler, and a collection vessel. Dry industrial residuals were ground into fine powder by means of a Pulverisette 11 blender (Fritsch, Hessen, Germany) for 30 s at 4000 g (powder diameter: 0.5–1 mm). Twenty grams of residuals were loaded in a 100 cm^3^ stainless steel vessel for supercritical fluid extractions. Restrictor temperature was set at 75 °C for each condition. The CO_2_ was pressurized in the vessel at a pressure of 380 bar and a temperature of 50 °C (d_CO2_ = 913 kg/m^3^). Once the set pressure was reached, a static period was maintained for 15 min; then, the valves were opened to collect the sample for 30 min in dynamic conditions (CO_2_ flow rate = 10 SCFH, standard cubic feet per hour, measured at the exit of the vessel, after CO_2_ expansion). Static and dynamic periods were chosen according to previous studies and preliminary experiments on the biomass. Identical extraction conditions were applied for 9 successive cycles.

##### 3.2.3. Supercritical CO_2_: Sequential Extraction

The same procedure described in Section 3.2.3 was used to perform the SFE sequential process. Twenty grams of pulverized residuals were loaded in the vessel for supercritical fluid extractions. Three experimental conditions corresponding to A: 35; 100 bar; 700 kg/m^3^, B: 45; 220 bar; 830 kg/m^3^, and C (equal to the single step): 50; 380 bar; 913 kg/m^3^ were applied one by one, keeping the material in the same vessel, to achieve complete extraction of oil components from Cp residuals. Pressures and temperatures were chosen in accordance with the results obtained in the single-step SFE process, in which the best conditions in terms of extraction yield were exploited to ensure complete exhaustion of the biomasses. The SFE process was divided into three sequential steps. Once the set pressure was reached, a static period was maintained for 15 min; then, the valves were opened to collect the sample for 30 min in dynamic conditions (CO_2_ flow rate = 10 SCFH).

### 3.3. Purification and Separation of Neutral and Acidic Fractions

Neutral and acid component separation was achieved by means of ion-exchange chromatography. Briefly, Amberlite IRA-400 was activated before use with sodium hydroxide solution, then water was removed by flushing methanol. Consequently, 50 mg of oil collected from each extraction were dissolved in methanol and added to 0.5 g of resin and kept under gentle magnetic stirring for 12 h to achieve the complete acid component adsorption. The mixture was then separated from the resin, and the non-adsorbed species were recovered by washing the resin with methanol. Retained fatty acids were eluted by adding a solution of acetic acid in methanol (1:1). Neutral components obtained from Cp residuals were then saponified following the Method Cd 3c–91/Method NGD C33-1976 [61]. Briefly, KOH ethanolic solution (2M) was added to Cp neutral fraction. The mixture was heated at boiling point for 2 h. After the saponification reaction, the obtained unsaponifiable matter was counter extracted in diethyl ether. The obtained mixture was washed three times with deionized water to remove the unreacted KOH and the saponified matter, and the solvent was evaporated under vacuum to weigh the resulting unsaponifiable fraction.

### 3.4. GC-MS Characterization

Before analyses, samples were derivatized by silylation following a procedure reported by Priestap et al. with some modifications. Briefly, 5 mg of dry extract were treated with 0.2 mL of N,O-Bis(trimethylsilyl)trifluoroacetamide (BSTFA) + 1% trimethylchlorosilane (TMCS) at 60 °C for 1 h [62]. Trimethylsilyl ether derivates were then analyzed by using an ISQTM QD Single Quadrupole GC-MS (Thermo Fisher, Waltham, MA, USA) equipped with a VF-5 ms (30 m × 0.25 mm i.d. × 0.25 µm; Agilent Technology, Santa Clara, CA, USA). Injection volume: 1 µL, split mode; oven program: 120 for 5 min; then 10 min^−1^ to 200; 5 min holding time; then 20 min^−1^ to 300; 20 min holding time; total run time: 38 min. Helium was used as a gas carrier (1 mL min^−1^) [62]. Ionization mode: electron impact: −70 eV. Acquisition mode: full scan. To identify the chemical structure of the eluted species, the fragmentation pattern of each peak was compared to the NIST 2014 database. The obtained solution was diluted 1:10 with 0.8 mL of ethyl acetate. NIST 2014 mass spectra library was used to identify isolated sterols and fatty acids in extracts.

Enzymatic reactions were monitored using an Agilent HP-5MS column (30 m × 0.25 mm × 0.25 μm) on a Finnigan TRACE DSQ GC-MS instrument (ThermoQuest, San Jose, CA). Elution conditions: 100 °C, 3 min; 20 °C min**^−^**^1^ to 300 °C then held for 1 min; inlet temperature: 300 °C; ion source temperature: 250 °C; MS transfer line temperature: 280 °C. Helium was used as a gas carrier (1 mL min^−1^). Ionization mode: electron impact: −70 eV. Acquisition mode: full scan. As described, the NIST 2014 database was used along with commercially available standards to identify the chemical structures of the eluates. Esterification and amidation reactions were analyzed without any further derivatization, while samples from biocatalyzed hydrations were analyzed after derivatization as methyl esters using a (trimethylsilyl)diazomethane solution in hexane: 30 μL of reactant of and 20 μL of MeOH were added to 100 mL of ethyl acetate extracts and briefly shaken at room temperature. Retention times (min): (a) ethyl esters: palmitic acid ethyl ester = 10.41, oleic/linoleic acid ethyl ester = 11.25, stearic acid ethyl ester = 11.35; (b) ethanol amides: palmitoyl ethanolamide = 12.82, oleoyl/linoleoyl ethanolamide = 13.60, stearoyl ethanolamide = 13.63; (c) methyl esters: capric acid methyl ester = 6.37, lauric acid methyl ester = 7.92, myristic acid = 9.15, palmitic acid = 10.25, oleic/linoleic methyl ester = 11.17, 10-hydroxy-*cis*-12-octadecenoic acid methyl ester = 11.86, 10-hydroxystearic acid methyl ester = 11.93.

### 3.5. Production and Purification of Recombinant Oleate Hydratase OhyA2

The gene coding for oleate hydratase OhyA2 (GenBank WP_017356052.1) from *Stenotrophomonas maltophilia* was synthetized and cloned in the pET28a vector in frame with a C-term His-tag sequence by Twist Bioscience (San Francisco, CA, USA), obtaining the plasmid pET28aSmOhyA2. *E. coli* BL21(DE3) was transformed with plasmid pET28aSmOhyA2 using standard techniques and stored at −80 °C as glycerol stock.

Enzyme expression was performed as follows. A single colony of *E. coli* BL21(DE3) carrying plasmid pET28aSmOhyA2 was inoculated in 100 mL of LB_KAN30_ and incubated for 24 h at 37 °C and 220 rpm. After that, the bacterial culture (20 mL) was inoculated and grown in 1 L of LB_KAN30_ culture medium at 37 °C and 220 rpm up to an OD_600_ of 0.6–0.8. Once reaching the desired OD_600_ value, the expression was induced by the addition of 0.2 mL of 0.5 M IPTG (0.1 mM final concentration) and the bacterial culture was incubated for a further 24 h at 16 °C and 200 rpm. The cells were harvested by centrifugation for 30 min at 4 °C and 5 000 rpm and resuspended in wash buffer (20 mM potassium phosphate (PB) buffer, pH 8.0, 500 mM NaCl, 20 mM imidazole). Then, the lysis of the bacterial cells was performed by ultrasonication (5 cycles of 30 s at 40% maximum power, Omni Ruptor 250-Watt Ultrasonic Cell Disrupter), followed by centrifugation for 30 min at 4 °C and 10 000 rpm. The protein purification was carried out by IMAC affinity chromatography, taking advantage of the His-tag at the protein C-terminal. As first step of purification, the cell lysate was collected and incubated on ice with Ni-NTA Sepharose 6 Fast Flow resin (GE Healthcare, Milan, Italy) for 90 min, then it was loaded onto a glass column (10 m × 110 m) previously equilibrated and washed with 15 mL of wash buffer. Protein elution was performed using a 3-step gradient (10 mL washing buffer containing 100, 200, and 300 mM imidazole, respectively). Subsequently, the protein fractions were collected and protein content was quantified using the Bradford method. Fractions containing the recombinant enzyme were dialyzed o/n at 4 °C against 50 mM citrate-PB buffer, pH 6.0, to remove residual salts and imidazole, and then stored at −80 °C.

### 3.6. Biotransformations

(*a*) *Lipase-mediated synthesis of fatty acid ethanol carboxamides*. Novozym^®^ 435 (2 g L**^−^**^1^) and 5Å molecular sieves were added to a solution of Cp oil (45 g L**^−^**^1^) prepared in a mixture of EtOH and cyclohexane (9:1, 5 mL final volume). The obtained mixture was incubated *in vacuo* at 55 °C and 160 rpm for 1 h. After assessing complete conversion by TLC (petroleum ether/EtOAc, phosphomolybdic reagent ((NH_4_)_6_MoO_4_ 42 g, Ce(SO_4_)_2_ 2 g, H_2_SO_4_ 62 mL, H_2_O 1 L) and GC-MS, the enzyme and molecular sieves were removed by filtration affording the ethyl ester derivatives after *in vacuo* concentration. The ester intermediates were than dissolved in ethanolamine (45 g L**^−^**^1^) and incubated at 75 °C and 160 rpm in the presence of 2 g L**^−^**^1^ Novozym^®^ 435 and 5Å molecular sieves for 72 h, monitoring their conversion via GC-MS. The target fatty acid ethanol carboxamides were recovered after filtration and *in vacuo* concentration.

(*b*) *Biocatalyzed hydration*. First, 0.1 g L**^−^**^1^ of OhyA2 were added to a solution of Cp oil (20 g L**^−^**^1^) prepared in 50 mM citrate-phosphate buffer, pH 6.5, containing 2% glycerol and EtOH, 0.15 mM FADH_2_ and titanium^III^ citrate (5 mM). After assessing complete conversion of oleic and linoleic acids by TLC (petroleum ether/EtOAc, phosphomolybdic reagent) and GC-MS (derivatization as methyl esters), pH was changed to 3 and the reaction mixture was extracted with EtOAc, recovering the transformed oil after drying over Na_2_SO_4_ and *in vacuo* concentration.

## 4. Conclusions

Sequential sc-CO_2_ extractions have been exploited on Cp residual biomass to achieve extracts enriched in selected target species.

Fractions with potential industrial interest have been obtained by means of supercritical fluids, mainly composed of free fatty acids and ∆7-sterols. Results obtained through sequential sc-CO_2_ technology underlined the feasibility of obtaining extracts with different compositions by tuning the physico-chemical parameters of the extraction (temperature and pressure), allowing the enrichment of target high-value classes of compounds. In particular, by treating under medium sc-CO_2_ conditions (in terms of pressure and temperature), the amount of extractables reached yields up to 8%, which is rewarding considering that the provided biomass was a “waste”.

The selected enzymatic manipulations investigated in this work and aimed at the future valorization of the valuable fatty acids still present in the considered industrial wastes could be carried out by exploiting very simple and scalable procedures, without any need for specific purification of the starting material, thus further demonstrating the applicability of biocatalysis in a circular economy view.

## Figures and Tables

**Table 1:** Main composition of extracts obtained from Cp seed residuals.

Entry	Extraction Conditions	Extraction Yield (%)	Free Fatty Acids (%)	Neutral Components (%)
1	Single step T = 50 °C; *p* = 380 bar d_CO2_ 913 kg/m^3^	10.8	14.5	Saponifiable 84.4 Unsaponifiable 1.1
2	(A) T = 35 °C; *p* = 100 bar d_CO2_ 700 kg/m^3^	1.2	71.0	Saponifiable 28.5 Unsaponifiable 0.5
3	(B) T = 45 °C; *p* = 220 bar d_CO2_ 830 kg/m^3^	7.5	23.6	Saponifiable 72.1 Unsaponifiable 4.1
4	(C) T = 50 °C; *p* = 380 bar d_CO2_ 913 kg/m^3^	2.8	4.2	Saponifiable 95.0 Unsaponifiable 0.8
	A + B + C	11.5		
5	*n*-Hexane	10.3	15.3	Saponifiable 83.6 Unsaponifiable 1.1

**Table 2:** Fatty acid relative abundances of CP extracts obtained.

Fatty Acids	Cp_sc-CO_2_/Single-Step Relative Abundance (%) *	Cp_*n*-Hexane Relative Abundance (%) *	Cp Oils from Literature Relative Abundance (%) [15,33,35,36,37,38,39,40,41,42,43,44] **
**Palmitic acid** (**16.0**)	11 ± 1	12.1 ± 0.5	8–15
**Linoleic acid** (**18.2**)	34 ± 1	33.6 ± 3	25–35
**Oleic acid** (**18.1**)	45 ± 2	44.7 ± 1	35–50
**Stearic acid** (**18.0**)	8.0 ± 0.4	8.4 ± 1	5–15
**Arachidic acid** (**20.0**)	0.7 ± 0.2	1.2 ± 0.1	0.5–1
**Saturated fatty acids**	19 ± 1	20 ± 2	15–25
**Monounsaturated fatty acids**	45 ± 1	45 ± 3	35–45
**Polyunsaturated fatty acids**	33 ± 2	34 ± 1	25–35

* Obtained after industrial extraction of Cp seeds. ** Data collected from untreated Cp seeds.

**Table 3:** Fragmentation ions used for identification of sterol trimethylsilyl ethers. [M^+^] molecular ion and [M−15]^+^ loss of the methyl terminal group; [M−90]^+^ corresponding to [M-(CH_3_)_3_SiOH]^+^; [M-105]^+^ corresponding to [M-(CH_3_)_3_SiOH-CH_3_]^+^.

Sterols	Main Fragmentation Ions, *m/z*				
	[M]^+^	[M-15]^+^	[M-90]^+^	[M-105]^+^	Sterol Abundance (%)
**β-** **Sitosterol**	486	471	396	381	1.6 ± 0.3
**Δ7,25-Stigmastadienol**	484	469	394	379	31 ± 2
**Δ7-Stigmastenol**	486	471	396	381	39 ± 2
**Δ7-Spinasterol**	484	469	394	379	27 ± 3
**Δ7,22,25-Stigmastatrienol**	482	467	392	377	4.1 ± 0.6
**Δ** **7-Avenasterol**	484	469	394	379	7 ± 1

## Data Availability

Not applicable.

## Supplementary Materials

The following supporting information can be downloaded at: https://www.mdpi.com/article/10.3390/molecules27154783/s1. Figure S1. Palmitic acid; Figure S2. Palmitic acid methyl ester; Figure S3. Palmitoyl ethanolamide; Figure S4. Linoleyl ethanolamide; Figure S5. Oleyl ethanolamide.

## Sample Availability

Samples of the compounds are not available from the authors.
