# Supplementary material for: Selective Supercritical CO2 Extraction and Biocatalytic Valorization of Cucurbita pepo L. Industrial Residuals
Source: Molecules. 2022 Jul 26;27(15):4783. doi: 10.3390/molecules27154783 (PMC9332722; doi:10.3390/molecules27154783)
Supplement: Supplementary file 1 [file molecules-27-04783-s001.zip › molecules-1817015-supplementary.pdf]

Supporting Information

GC/MS analyses of the biocatalytic esterification and amidation of palmitic acid (used for optimization) and of the ethanol amides of oleic and linoleic acids prepared as standards

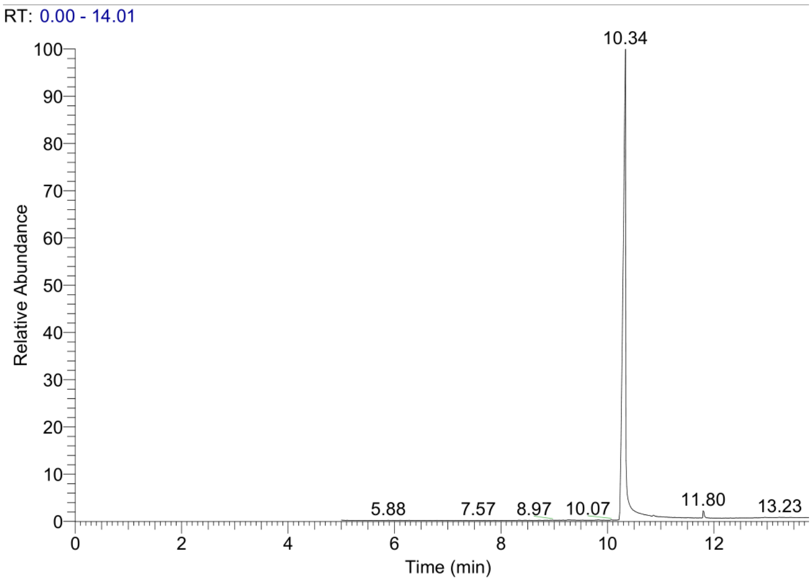

Figure S1. Palmitic acid

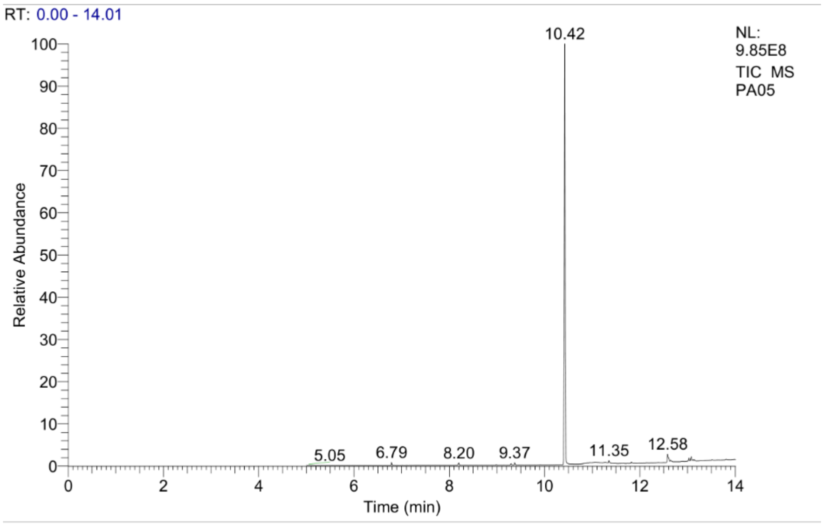

Figure S2. Palmitic acid methyl ester

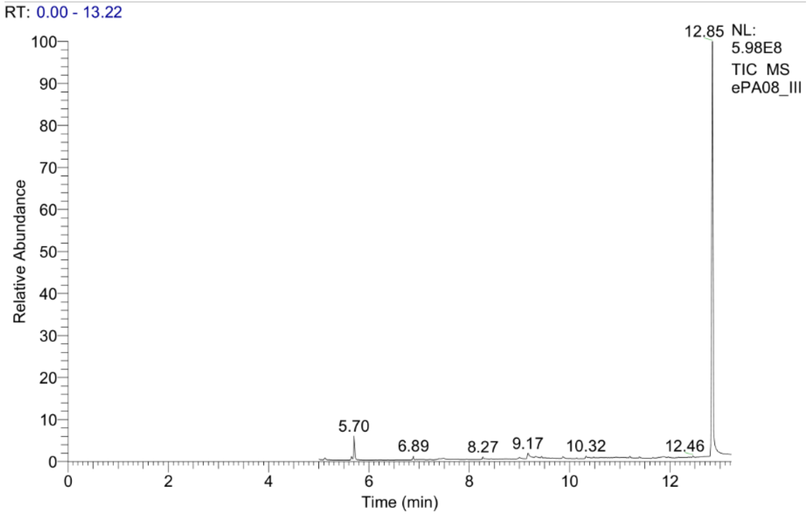

Figure S3. Palmitoyl ethanolamide

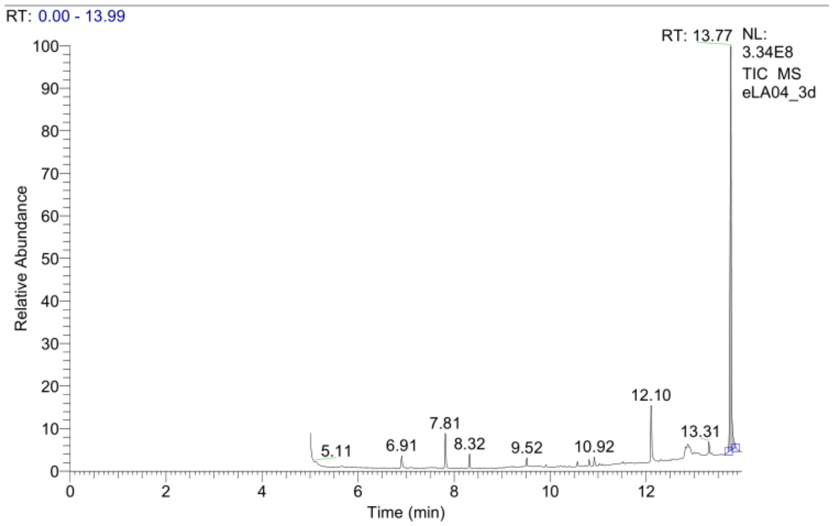

Figure S4. Linoleyl ethanolamide

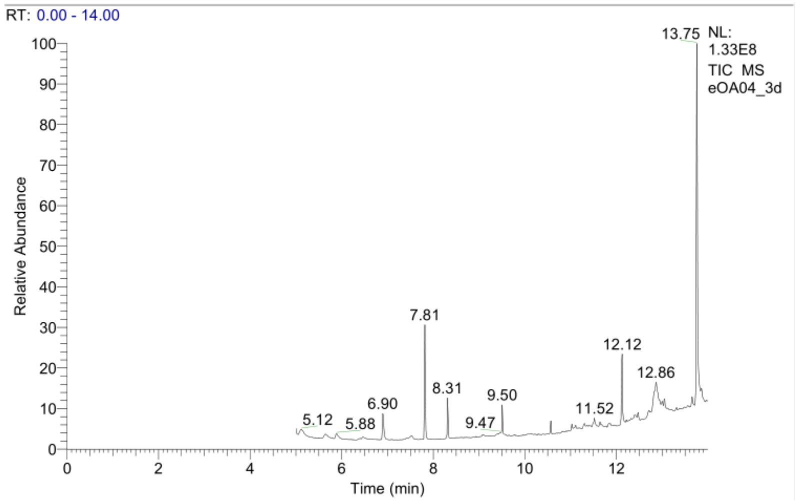

Figure S5. Oleyl ethanolamide
